# Gender differences in the relationship between alcohol consumption and insomnia in the northern Chinese population

**DOI:** 10.1371/journal.pone.0207392

**Published:** 2018-12-06

**Authors:** Yuchen Guo, Hongpu Hu, Yingping Liu, Yue Leng, Xing Gao, Qinghua Cui, Jianxin Chen, Bin Geng, Yong Zhou

**Affiliations:** 1 Department of Health Information Management, Institute of Medical Information, Chinese Academy of Medical Sciences & Peking Union Medical College, Beijing, China; 2 Beijing Obstetrics and Gynecology Hospital, Capital Medical University, Beijing, China; 3 Department of Psychiatry, University of California, San Francisco, California, United States of America; 4 Department of Biomedical Informatics, School of Basic Medical Sciences, Key Laboratory of Molecular Cardiovascular Science of the Ministry of Education Center for Non-coding RNA Medicine, Peking University Health Science Center, Beijing, China; 5 Beijing University of Chinese Medicine, Beijing, China; 6 Hypertension Center, Fuwai Hospital, Chinese Academy of Medical Sciences and Peking Union Medical College, State Key Laboratory of Cardiovascular Disease, Beijing, China; 7 Sanbo Brain Institute, Sanbo Brain Hospital, Capital Medical University, Beijing, China; Edith Cowan University, AUSTRALIA

## Abstract

**Background:**

Insomnia is one of the main symptoms of sleep disorders. Previous studies have suggested that alcohol intake is associated with several adverse health outcomes. The association between alcohol consumption and insomnia has been addressed in several studies with different results. However, whether gender may modify the association between alcohol consumption and insomnia is not clear. This study will focus on gender differences in the relationship between alcohol consumption and insomnia.

**Methods:**

The final study includes 8081 subjects aged between 18 and 65 years from the Jidong cohort. The data on alcohol consumption is collected by questionnaires, and insomnia problems are assessed using the entire 8-item Athens Insomnia Scale (AIS-8). Logistic analysis is used to evaluate the association between alcohol consumption and insomnia.

**Results:**

Among the 8081 participants in this study, 2618 (32.4%) are alcohol drinkers, including 2424 males and 194 females. The prevalence of insomnia is 9.6% in the male and 10.6% in the female. The adjusted odds ratios (ORs) with 95% confidence interval (CI) of mild-to-moderate drinkers and heavy drinkers for insomnia are 1.27 (1.02–1.58) and 1.02 (0.79–1.32), respectively. Heavy alcohol consumption is significantly correlated with insomnia in the female, after controlling for potential confounding factors (OR: 2.11, 95% CI: 1.28–3.49, *p* for interaction = 0.002).

**Conclusion:**

A significant association between alcohol consumption and insomnia is found in females, but not in males from the northern Chinese population.

## Introduction

Alcohol consumption has created various global public health problems. There are now nearly two billion alcohol drinkers around the world [1]. The WHO global status on alcohol and health reports that more than half of the population aged 15 years and older in China are alcohol consumers [2]. Alcohol consumption is associated with a variety of psychiatric disorders, especially depression and sleep disorders [3, 4]. Insomnia is one of the main symptoms of sleep-related disturbances, which is considered to be a common clinical condition characterized by difficulty in initiating or maintaining sleep. The prevalence of insomnia is ranging from 6% to 33% in the general population [5].

Previous studies have attempted to identify the possible modifiable factors which could potentially help people to sleep. Alcohol is used as a hypnotic to help those with insomnia to fall asleep [6]. The currently available data also suggest that sleep effects actually appear to be associated with the reinforcement effects of ethanol as a hypnotic for insomniacs [7–9]. However, some laboratory studies have reported that alcohol quickly loses its effectiveness as a hypnotic while retaining its sleep disturbing properties after following chronic consumption [10, 11]. Notably, the gender difference may alter the association between alcohol use and insomnia, and it has been extensively studied in some researches [12, 13]. For example, a study based on Brazilian subjects showed that subjective sleep disturbance was prevalent in all women (100%) and most of men (88.9%) after drinking. On the one hand, men are more likely to carry the endogenous vulnerabilities and exogenous risks that increase their sleep problems due to alcohol dependence. On the other hand, women might be more susceptible to the effects of alcohol consumption than men because of the higher blood alcohol concentrations after drinking equivalent doses of alcohol per kilogram of body weight [14, 15]. This study hereby hypothesizes that the relationship between alcohol consumption and insomnia differs from gender to gender. Therefore, the aim of this study is to investigate whether gender differences will modify the association between alcohol consumption and insomnia in the northern Chinese population.

## Materials and methods

### Study design and population

We used data from Jidong cohort, which was a community-based cohort for prospective researches on Chinese adults. Jidong community is located in Tangshan, which is a large and modern industrial city in the central section of the Bohai Rim in HeBei Province, of northern China [16]. From July 2013 to August 2014, a total of 9078 residents aged ≥ 18 years in Jidong community were recruited to participate in the study. Inclusion and exclusion criteria have been demonstrated in previous publication [17, 18]. In brief, all candidates who successfully replied to a standardized questionnaire were selected. 19 subjects with missing data on alcohol consumption, 714 subjects with incomplete information of Athens Insomnia Scale (AIS-8), and 264 subjects with a history of cardiovascular disease (such as atrial fibrillation, heart failure, myocardial infarction and so on) or cancer were all excluded. Finally, 8081 participants were included in this study.

The study was performed according to guidelines from the Helsinki Declaration. Ethical approvals were obtained from the Ethics Committee of Jidong Oil- field, Inc. Medical Centers. All participants signed informed consent.

### Measurement of alcohol consumption

Data on alcohol consumption were collected using questionnaires [17, 19, 20]. The questionnaires gathered information on the drinking frequency and the average amount of alcohol consumed per day. Participants were also asked to report the number of standard drinks. The total alcohol intake was calculated in grams by multiplying drinking frequency per day, the average amount of alcohol consumed and the volume of alcohol contained (the volume of ethanol is 12.5% in wine, 5% in beer and 45% in liquor). In this study, 15g of ethanol was taken as a standard serving [21]. On the basis of the number of standard servings, participants were classified into three categories: none (never drank or drank in the past), mild-to-moderate (women ≤1.0 servings/day; men ≤2.0 servings/day) and heavy (women >1.0 servings/day; men > 2.0 servings/day) [22].

### Assessment of insomnia

The entire 8-item Athens Insomnia Scale (AIS-8) is a self-assessment psychometric instrument based on the Tenth Revision (ICD-10) diagnostic criteria, which has been developed as a tool to evaluate the severity of insomnia. In addition, AIS-8 is not only used to measure the intensity of sleep difficulty reliably, but also to assist in establishing the diagnosis of insomnia, and thus it has become an invaluable tool in sleep research and clinical practice. In this study, the AIS-8 was created into a questionnaire including 8 items. The first 5 items were used to assess the difficulty with sleep induction, awakenings during the night, early morning awakening, total sleep time, and the overall quality of sleep. While another 3 items were used to evaluated the consequences of insomnia on the following day, including sense of well-being, functioning, and sleepiness during daytime [23]. The entire 8-items AIS had a total score ranging from 0 to 24, with each item rating from 0 to 3. Participants with 0 score corresponded to “no problem at all”, and those with 3 scores corresponded to “very serious problem”. Subjects with AIS scores ≥6 were recognized as insomnia victims [24].

### Assessment of covariates

Data on demographic (age, sex, marital status, education level, income, physical activity and smoking status) and clinical characteristics (body mass index (BMI), hypertension, diabetes and insomnia) were collected via questionnaires. The levels of education were classified into three categories: primary school and below, middle and high school, college and above. The physical activities of participants were divided into three categories according to the following three kinds of circumstances: (1) inactive, nearly none; (2) moderately active, 1–149 min/ week of moderate intensity or 1–74 min/week of vigorous intensity; or (3) active, ≥150 min/week of moderate intensity or ≥75 min/week of vigorous intensity [25]. The smoking status were classified as never (<100 cigarettes in the entire life), past or current smoker. After measuring body weight (kg) and height (cm) for each subject on the day of tests, the BMI was calculated as body weight (kg) divided by the square of height (m) [26]. Simultaneously, hypertension was defined as a self-reported history of hypertension, including the use of antihypertensive medication, systolic blood pressure ≥140 mm Hg or diastolic blood pressure ≥90 mm Hg [26, 27]. The definition of diabetes mellitus was fasting blood glucose ≥7.0 mmol/L, current treatment with insulin/oral hypoglycemic agents or a history of diabetes mellitus [28].

All biochemical variables were measured at the central laboratory at Jidong Oilfield Hospital, including total cholesterol (TC was measured using the endpoint test method), triglyceride (TG was measured using the GPO method), high-density lipoprotein cholesterol and low-density lipoprotein cholesterol (HDL-C and LDL-C levels were measured using the direct test method) [17].

### Statistical analysis

Statistical analysis was performed with SAS software, version 9.4 (SAS Institute, Cary, North Carolina, USA). Normal distribution of continuous variables was tested using the Kolmogorov-Smirnov test. The continuous data underlying normal distribution were presented as mean values (standard deviation, SD) and compared via T-test or ANOVA analysis; While non-normal distribution variables were presented as median values (interquartile range, IQR) and compared using corresponding nonparametric methods. Categorical variables were presented as counts and percentages, and compared using chi-squared tests or the Fisher’s exact test, when appropriate. Logistic regression analyses were performed to assess the association between alcohol consumption and insomnia by calculating the odds ratios (ORs) and 95% confidence interval (95% CI), with the adjustments for age, sex, income, BMI, education level, smoking status, physical activity, hypertension, diabetes, TC, TG, HDL-C and LDL-C. Furthermore, this study not only evaluated the relationship between alcohol consumption and insomnia among all participants, but also stratified the data by gender and age.

All statistical analyses were 2-sided and the *P*-value <0.05 was considered statistically significant.

## Results

### Baseline characteristics

**Fig 1:**
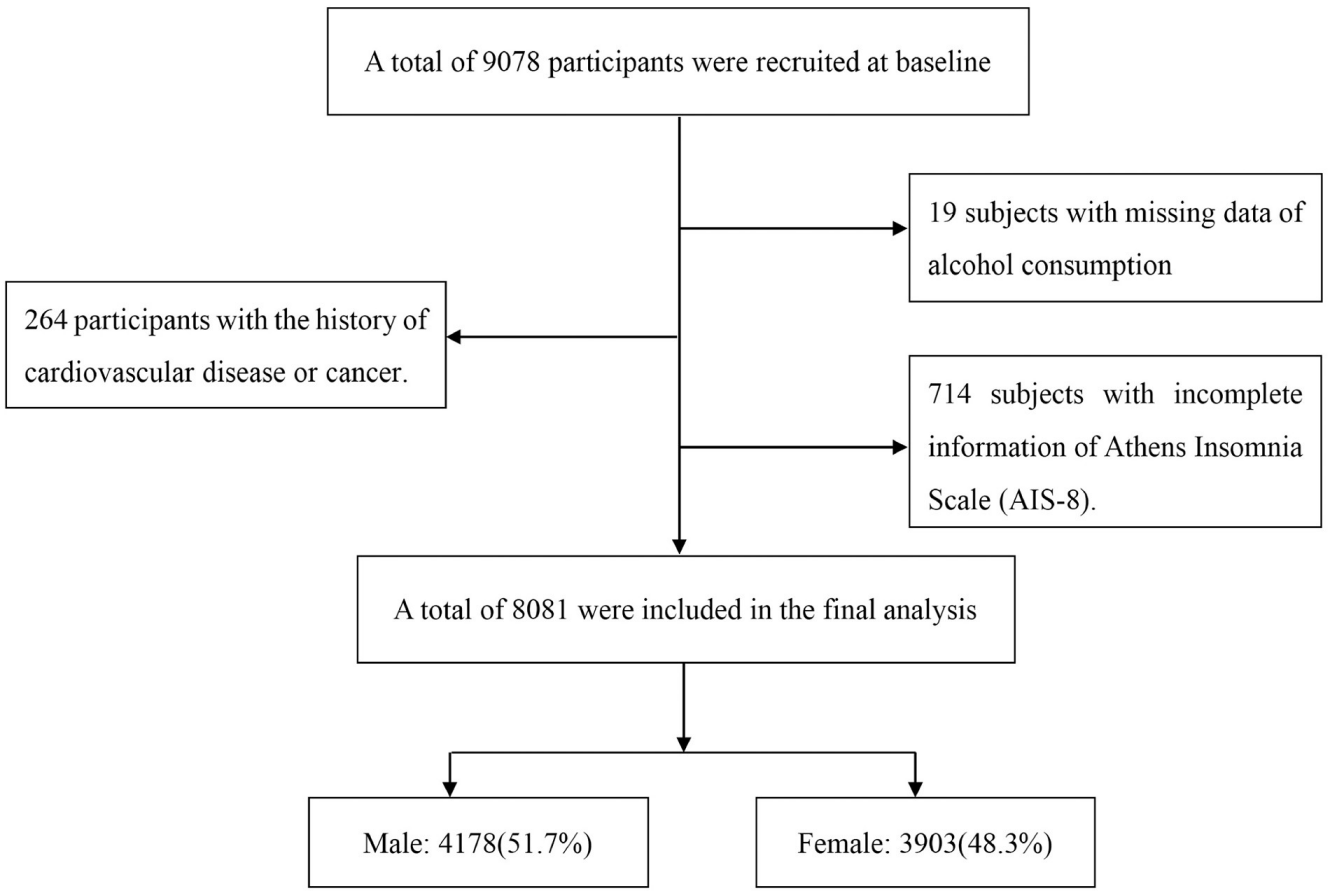
Flow chart of subjects with different exclusion criteria in this study.

Fig 1 shows the flow chart of this study. Totally, 9078 residents were selected, among which 8081 of them were recruited in the present study. The baseline characteristics of alcohol consumption are shown in Table 1. Among 8081 eligible subjects, 2618 (32.4%) are alcohol drinkers (mild-to-moderate drinkers: 59.1%; heavy drinkers: 40.9%), including 2424 (92.6%) males and 194 (7.4%) females. Alcohol consumers have a relatively higher income and education level than non-drinkers, with 52.8% (1381/2618) current smokers. In addition, mild-to-moderate and heavy alcohol drinkers also have a higher prevalence of hypertension than non-drinkers (44.5% vs 31.1% and 45.5% vs 31.1%, respectively).

**Table 1:** Baseline characteristics of total alcohol consumption.

Characteristic	Total (N = 8081)	Alcohol consumption			*P*-value
		None (n = 5463)	Mild-to-Moderate (n = 1546)	Heavy (n = 1072)	
**Age, year**	42.1±13.0	42.8±13.3	41.5±12.5	39.7±11.5	<0.01
**Male, n (%)**	4178 (51.7)	1754 (32.1)	1446 (93.5)	978 (91.2)	<0.01
**Married, n (%)**	7511 (93.0)	5104 (93.4)	1426 (92.2)	981 (91.5)	0.04
**BMI (SD), kg/m**^**2**^	24.5±3.7	24.1±3.7	25.5±3.5	25.5±3.6	<0.01
**Income**					<0.01
≤3000, n (%)	3066 (37.9)	2211 (40.5)	560 (36.2)	295 (27.5)	
3000–5000,n (%)	4400 (54.5)	2914 (53.3)	829 (53.6)	657 (61.3)	
>5000, n (%)	615 (7.6)	338 (6.2)	157 (10.2)	120 (11.2)	
**Education level**					<0.01
Primary school and below, n (%)	296 (3.7)	241 (4.4)	34 (2.2)	21 (2.0)	
Middle and high school, n (%)	2824 (34.9)	2038 (37.3)	500 (32.3)	286 (26.7)	
College and above, n (%)	4961 (61.4)	3184 (58.3)	1012 (65.5)	765 (71.4)	
**Physical activity**					<0.01
Inactive, n (%)	3118 (38.6)	2186 (40.0)	559 (36.2)	373 (34.8)	
Moderately active, n (%)	769 (9.5)	481 (8.8)	171 (11.1)	117 (10.9)	
Active, n (%)	4194 (51.9)	2796 (51.2)	816 (52.8)	582 (54.3)	
**Smoking status**					<0.01
Never, n (%)	5734 (71.0)	4663 (85.4)	626 (40.5)	445 (41.5)	
Current, n (%)	2068 (25.6)	687 (12.6)	817 (52.9)	564 (52.6)	
Past, n (%)	279 (3.4)	113 (2.1)	103 (6.7)	63 (5.9)	
**Diabetes, n (%)**	524 (6.5)	344 (6.3)	106 (6.9)	74 (6.9)	0.61
**Hypertension, n (%)**	2881 (35.7)	1700 (31.1)	704 (45.5)	477 (44.5)	<0.01
**TG (SD), mmol/L**	1.6±1.3	1.4±1.1	2.0±1.8	1.9±1.5	<0.01
**TC (SD), mmol/L**	4.5±0.9	4.4±0.9	4.5±0.9	4.5±0.9	<0.01
**HDL-C (SD), mmol/L**	1.2±0.3	1.2±0.3	1.1±0.3	1.1±0.2	<0.01
**LDL-C (SD), mmol/L**	2.5±0.6	2.5±0.6	2.5±0.6	2.6±0.6	<0.01
**Insomnia, n (%)**	1028 (12.7)	757 (13.9)	176 (11.4)	95 (8.9)	<0.01

Values are expressed as mean value ± SD, median value (IQR), or percentage. TG = Triglyceride; TC = Total Cholesterol; HDL-C = High-density lipoprotein cholesterol; LDL-C = Low-density lipoprotein cholesterol; SD = standard deviation.

### Prevalence of insomnia

**Fig 2:**
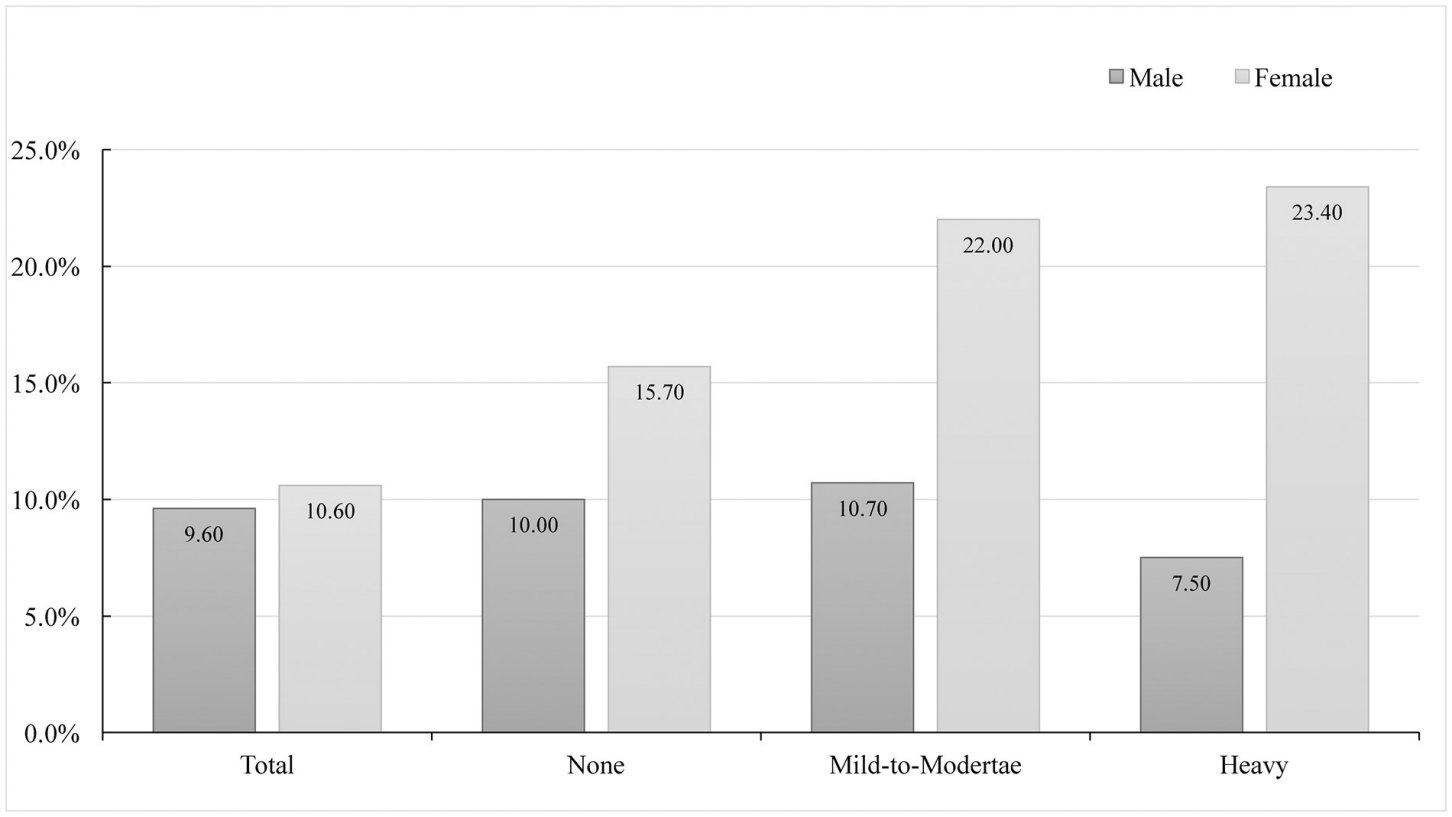
The prevalence of insomnia between males and females according to alcohol consumption.

The prevalence of insomnia among non-drinkers, mild-to-moderate drinkers and heavy drinkers are shown in Fig 2. The prevalence of insomnia is 12.7% among the whole participants, and it is higher in females (16.0%) than in males (9.6%). Among the males, the prevalence of insomnia is 10.0% in non-drinkers, 10.7% in mild-to-moderate drinkers and 7.5% in heavy drinkers. Whereas among females, the prevalence of insomnia increases along with the increase of alcohol intake, which is 15.7% in none group, 22.0% in mild-to-moderate group and 23.4% in heavy group.

### Association between alcohol consumption and insomnia

**Fig 3:**
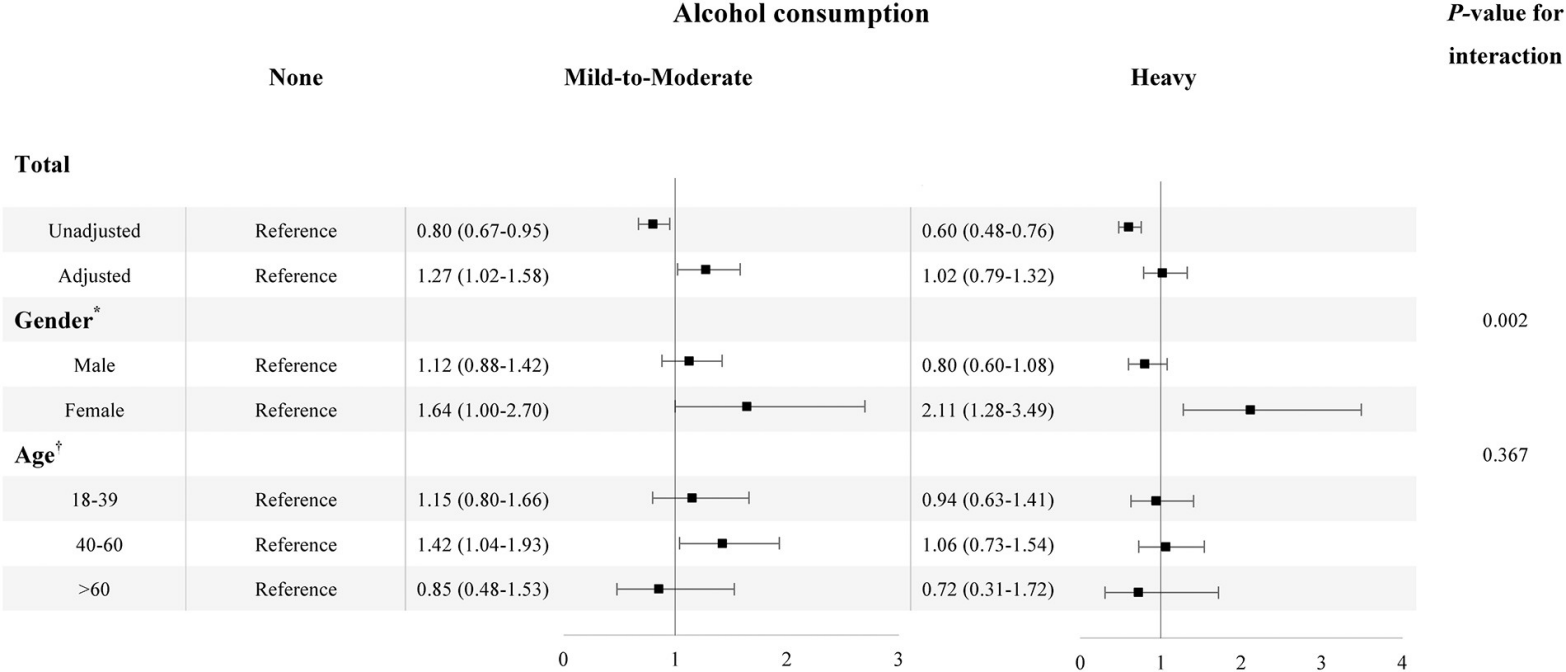
Odds radio with 95% CI of alcohol consumption on insomnia in total, different gender and age categories. For the total subjects, we presented the association without the introduction of the confounding factors, and we also presented the association controlling for age, gender, BMI, income, education level, physical activities, smoking status, hypertension, diabetes mellitus, TC, TG, HDL-C and LDL. In the stratification analysis, all the covariates were controlled.

Fig 3 demonstrates the association between insomnia and alcohol consumption in the whole participants, and stratifies by age and gender. After adjusting for age, gender, BMI, income, education level, physical activities, smoking status, hypertension, diabetes, TC, TG, HDL-C and LDL-C, the association between mild-to-moderate alcohol consumption and insomnia is statistically significant (OR:1.27, 95% CI:1.02–1.58). The *P* value for interaction about gender difference in the association between alcohol consumption and insomnia is statistically significant (*P*_inter_ = 0.002). Thus, a positive correlation between heavy alcohol consumption and insomnia is found in the female (OR:2.11, 95% CI:1.28–3.49), but not in the male (OR:0.80, 95% CI:0.60–1.08). Stratified by age, the mild-to-moderate alcohol consumption is associated with insomnia among participants aged above 45 years (OR:1.44, 95% CI:1.07–1.92), though the interaction is not statistical significance (*P*_inter_ = 0.367).

## Discussion

This large community-based study shows that alcohol consumption is significantly associated with insomnia in females, but not in males. Additionally, it is the first attempt to demonstrated a diverse relationship on gender between alcohol consumption and insomnia in the northern Chinese population.

Several previous studies suggested that there was a relationship between alcohol consumption and insomnia. Consistent with our findings, some studies have found that alcohol consumption might be a risk factor for sleep disorders in general population [29, 30]. Hartwell et al demonstrated a robust association between alcoholism severity and sleep disorders determined by the Pittsburgh Sleep Quality Index (PSQI), and concluded that the problem of alcohol severity might be a predictive signal of sleep disorders [31]. In a 10-year prospective study (N = 2602), Janson and the colleagues found that some subsequent insomnia symptoms were associated with alcohol dependence (moderate-to-severe alcohol consumption disorder), with insomnia assessed by CAGE questionnaire [32]. Similarly, two studies performed in men showed that alcohol use could prevent subjects initiating sleep or make them hard of falling asleep [33, 34]. In addition, some literature also reported that the relationship between alcohol and sleep appeared to be dose related, stating that alcohol consumption might have stimulant effects when it was absorbed at low to moderate dose, and have sedative effects at higher dose and during alcohol elimination [35]. A study found that there was a positive relationship between mild-to-moderate doses alcohol consumption and insomnia in this large gender-based sample population, which was inconsistent with those previous detections. Different results about the relationship between alcohol consumption and insomnia might occur due to several possible reasons: (1) The lack of standard definitions and measurements for both alcohol consumption and insomnia; (2) The different population distribution and sample sizes were not taken into account; (3) There were differences in alcohol pharmacokinetics between men and women.

In the present study, the major finding is that alcohol consumption is associated with insomnia in females, but not in males. Female heavy drinkers have a significantly higher risk of suffering from insomnia than their counterpart males. A lot of evidences have shown an apparent difference in alcohol consumption between males and females [13, 36]. As we all know, the gender gap in alcohol drinking is one of the universal gender differences in human social behavior, which also reflects a different effect on drinking men and women in different drinking cultures. In China, alcohol consumption was generally not discouraged for women, while it was widely accepted in men in the traditional Chinese culture [37]. There were also some obvious differences in the numbers of drinkers and the frequency of alcohol consumption among men and women [8, 38]. The gender difference in the relationship between alcohol consumption and insomnia might be ascribed to alcohol pharmacokinetics. A study based on 93 healthy adults (females: 59; males: 34) found that alcohol objectively disrupted sleep continuity more in the female than in the male only at equally high peak breath alcohol concentrations (BrAC) throughout the entire night [39]. Compared to men, women were likely to suffer more psycho-social and mental impairments due to alcohol intake. Accordingly, the reasons for this result are multifactorial and multilevel, which suggest for further consideration of gender differences in the future research. In addition, we have found that mild-to-moderate alcohol consumption is associated with insomnia in participants aged above 45 years, though the interaction is not statistically significant, which indicates that alcohol intake might be a risk for older individuals. Inconsistent with our finding, a study based on a large proportion of alcoholics detected that only older age was associated with improvements in sleep disturbances during early alcohol recovery [40]. The possible reasons for these different conclusions might be ethnic or gender differences.

Furthermore, some previous studies examined the biological, psychological and social risk factors for the association between alcohol consumption and insomnia, though only few of them explained the mechanisms in the correlation of alcohol use and sleep disorders. Based on limited literature and research results, we have presented several possible mechanisms of the association between alcohol consumption and insomnia for females. On the one hand, the prevalence of mental and psychological illness was higher in females than that in males, especially depression and anxiety. Alcohol intake could stimulate the physiological sensitivity of women, which might help explain the high prevalence of insomnia among women [41]. In addition, since alcohol had a stimulating effect on nervous system, alcohol might motivate the female brain to make them excited, while damaging their neurons, and making them hard of initiating sleep [42]. On the other hand, the primary insomnia was associated with menstrual-related changes in the female, and these menstrual-related changes might affect subjective and objective sleep disorders, especially in the late luteal phase [43, 44]. This evidence has shown that estrogen plays an important regulatory role for female insomnia. Alcohol consumption might have an effect on female estrogen hormone that also led to insomnia.

There are several limitations in this study. Firstly, this study is a cross-sectional study, making it unable to explore the causal effect between insomnia and alcohol consumption. Secondly, the Athens Insomnia Scale (AIS-8) is not used for clinical diagnosis of insomnia, even though it is a widely used epidemiological tool for insomnia assessment. Thirdly, the study is based on the self-reported data and absence of objective measures against insomnia such as polysomnography (PSG) or actigraphy, and the biological markers of alcohol consumption or breath alcohol tests are absent.

## Conclusion

This study suggests that alcohol consumption is associated with insomnia in females, but not in males. To a certain extent, alcohol consumption may prevent females from initiating asleep.

## Supporting information

S1 Table Baseline characteristics of different alcohol consumption groups between male and female. (DOCX)
