# Supplementary material for: Gender differences in the relationship between alcohol consumption and insomnia in the northern Chinese population
Source: PLoS One. 2018 Dec 6;13(12):e0207392. doi: 10.1371/journal.pone.0207392 (PMC6283629; doi:10.1371/journal.pone.0207392)
Supplement: S1 Table — (DOCX) [file pone.0207392.s001.docx]

**Supporting Information**

**S1 Table. Baseline characteristics of different alcohol consumption groups between male and female.**

|  | **Alcohol Consumption** | | | | | | | | | |
| --- | --- | --- | --- | --- | --- | --- | --- | --- | --- | --- |
|  | **Male** | | | | | **Female** | | | | |
| **Characteristics** | **Overall (N=4178)** | **None (N=1754)** | **Mild-to-Moderate (N=1446)** | **Heavy (N=978)** | **P-value** | **Overall (N=3903)** | **None (N=3599)** | **Mild-to-Moderate (N=100)** | **Heavy (N=94)** | **P-value** |
| **Age (SD), year** | 41.7±13.1 | 43.2±14.0 | 41.4±12.7 | 39.7±11.7 | <0.01 | 42.5±12.8 | 42.6±12.9 | 42.4±10.4 | 39.3±9.8 | 0.05 |
| **Married, n (%)** | 3819(91.4) | 1596(91.0) | 1329(91.9) | 894(91.4) | 0.65 | 3692(94.6) | 3508(94.6) | 97(97.0) | 87(92.6) | 0.39 |
| **BMI (SD), kg/m^2^** | 25.5±3.5 | 25.4±3.6 | 25.6±3.5 | 25.6±3.6 | 0.11 | 23.4±3.6 | 23.4±3.6 | 23.4±3.4 | 23.6±3.9 | 0.89 |
| **Income** |  |  |  |  | <0.01 |  |  |  |  | <0.01 |
| ≤3000, n (%) | 1413(33.8) | 624(35.6) | 519(35.9) | 270(27.6) |  | 1653(42.4) | 1587(42.8) | 41(41.0) | 25(26.6) |  |
| 3000-5000,n (%) | 2392(57.3) | 1011(57.6) | 784(54.2) | 597(61.0) |  | 2008(51.5) | 1903(51.3) | 45(45.0) | 60(63.8) |  |
| >5000, n (%) | 373(8.9) | 119(6.8) | 143(9.9) | 111(11.4) |  | 242(6.2) | 219(5.9) | 14(14.0) | 9(9.6) |  |
| **Education level** |  |  |  |  | <0.01 |  |  |  |  | 0.05 |
| Primary school and below, n (%) | 110(2.6) | 62(3.5) | 28(1.9) | 20(2.0) |  | 186(4.8) | 179(4.8) | 6(6.0) | 1(1.1) |  |
| Middle and High school, n (%) | 1311(31.38) | 597(34.0) | 455(31.5) | 259(26.5) |  | 1513(38.8) | 1441(38.9) | 45(45.0) | 27(28.7) |  |
| College and above, n (%) | 2757(66.0) | 1095(62.4) | 963(66.6) | 699(71.5) |  | 2204(56.5) | 2089(56.3) | 49(49.0) | 66(70.2) |  |
| **Physical activity** |  |  |  |  | 0.19 |  |  |  |  | 0.15 |
| Inactive, n (%) | 1491(35.7) | 630(35.9) | 520(36.0) | 341(34.9) |  | 1627(41.7) | 1556(42.0) | 39(39.0) | 32(34.0) |  |
| Moderately active, n (%) | 426(10.2) | 158(9.0) | 161(11.1) | 107(10.9) |  | 343(8.8) | 323(8.7) | 10(10.0) | 10(10.6) |  |
| Active, n (%) | 2261(54.1) | 966(55.1) | 765(52.9) | 530(54.2) |  | 1933(49.5) | 1830(49.3) | 51(51.0) | 52(55.3) |  |
| **Smoking status** |  |  |  |  | <0.01 |  |  |  |  | <0.01 |
| Never, n (%) | 1896(45.4) | 994(56.7) | 538(37.2) | 364(37.2) |  | 3838(98.3) | 3669(98.9) | 88(88.0) | 81(86.2) |  |
| Current, n (%) | 2007(48.0) | 650(37.0) | 805(55.7) | 552(56.4) |  | 61(1.6) | 37(1.0) | 12(12.0) | 12(12.8) |  |
| Past, n (%) | 275(6.6) | 110(6.3) | 103(7.1) | 62(5.4) |  | 4(0.1) | 3(0.1) | 0(0) | 1(1.0) |  |
| **Diabetes, n (%)** | 335(8.0) | 162(9.2) | 100(6.9) | 73(7.5) | 0.04 | 189(4.8) | 182(4.9) | 6(6.0) | 1(1.1) | 0.20 |
| **Hypertension, n (%)** | 1664(39.8) | 691(39.4) | 578(40.0) | 395(40.4) | 0.87 | 997(25.5) | 951(25.6) | 26(26.0) | 20(21.3) | 0.63 |
| **TG(SD), mmol/L** | 1.9±1.5 | 1.7±1.2 | 2.0±1.9 | 1.9±1.5 | <0.01 | 1.3±1.0 | 1.3±1.0 | 1.3±0.9 | 1.3±0.9 | 0.79 |
| **TC(SD), mmol/L** | 4.5±0.9 | 4.4±0.9 | 4.5±0.9 | 4.5±0.9 | 0.01 | 4.4±0.9 | 4.4±0.9 | 4.4±0.8 | 4.3±0.8 | 0.39 |
| **HDL-C(SD), mmol/L** | 1.1±0.2 | 1.1±0.2 | 1.1±0.2 | 1.1±0.2 | <0.01 | 1.3±0.3 | 1.3±0.3 | 1.3±0.3 | 1.3±0.3 | 0.14 |
| **LDL-C(SD), mmol/L** | 2.6±0.6 | 2.5±0.6 | 2.6±0.6 | 2.6±0.6 | 0.23 | 2.4±0.6 | 2.4±0.6 | 2.4±0.6 | 2.3±0.5 | 0.41 |

Values are expressed as mean value ± SD, median value (IQR), or percentage.

TG = Triglyceride; TC = Total Cholesterol; HDL-C = High-density lipoprotein cholesterol; LDL-C = Low-density lipoprotein cholesterol; SD = standard deviation.
